# Frailty and its combined effects with lifestyle factors on cognitive function: a cross-sectional study

**DOI:** 10.1186/s12877-023-03761-0

**Published:** 2023-02-06

**Authors:** Fangqing Li, Yike Yan, Lei Zheng, Chenming Wang, Xin Guan, Shiru Hong, Huan Guo

**Affiliations:** Department of Occupational and Environmental Health, State Key Laboratory of Environmental Health (Incubating), School of Public Health, Tongji Medical College, Huazhong University of Science and Technology, 13 Hangkong Rd, Wuhan, 430030 Hubei China

**Keywords:** Frailty, Mini-mental state examination, Cognitive function, Lifestyles

## Abstract

**Background:**

Frailty describes an age-related state of deterioration in biological function. This study aimed to investigate the association between frailty and cognitive function and its combined effects with lifestyles.

**Methods:**

A total of 3,279 participants from the Dongfeng-Tongji (DFTJ) cohort were tested the cognitive function by using the Chinese version of Mini-mental State Examination (MMSE). Frailty was evaluated based on a 35-item frailty index (FI). Frailty status was dichotomized into robust (FI < 0.15) and frail (FI ≥ 0.15). Multivariate generalized linear regression models and logistic regression models were used to estimate the associations of frailty with MMSE score and cognitive impairment. We also analysed the modification and combined effects of lifestyle factors, including smoking status, drinking status, and regular physical exercise, on the above associations.

**Results:**

FI was significantly associated with lower MMSE score [β (95%Cl) = -0.28 (-0.43, -0.13)] and cognitive impairment [OR (95%Cl) = 1.19 (1.04, 1.35)]. The association of frailty status with MMSE were found to be stronger among ever smokers [β(95%Cl) = -1.08 (-1.64, -0.51)] and physical inactive individuals [β(95%Cl) = -1.59 (-2.63, -0.54)] while weaker or not significant among never smokers [β(95%Cl) = -0.30 (-0.62, 0.01)] and physical active individuals [β(95%Cl) = -0.37 (-0.65, -0.08))]. There were significant combined effects of frailty status with unhealthy lifestyles including smoking, alcohol drinking, and physical inactive on cognitive impairment.

**Conclusions:**

Frailty was associated with cognitive impairment among Chinese middle-aged and elderly people, while smoking cessation and regular physical exercise could attenuate the above associations, which highlight the potential preventive interventions.

**Supplementary Information:**

The online version contains supplementary material available at 10.1186/s12877-023-03761-0.

## Background

With the accelerating process of global population aging, cognitive impairment is becoming a critical issue worldwide. Recent study demonstrated that the prevalence of mild cognitive impairment (MCI), an intermediate stage between normal aging and dementia [1], increased from 6.7% for subjects aged 60–64 to 25.2% for those aged 80–84 [1]. Although MCI may revert back to a normal cognitive state, about one-third MCI patients develop Alzheimer's disease (AD) within 5 years [2]. The Global Burden of Disease Study 2016 reported a 28.8 million loss of disability adjusted life years (DALYs) due to dementia, which brought huge burdens to the society and health-care system [3]. In particular, more than 11 million caregivers provided an estimated 16 billion hours of care to dementia patients with an unpaid dementia caregiving equivalent to $271.6 billion in 2021. Moreover, its costs extended to family caregivers’ increasing risk for emotional suffering, as well as adverse mental and physical health outcomes [4]. However, practical treatment for MCI and effective cure for dementia are absent at present [5]. Therefore, discovering potential modifiable risk factors and implementing primary prevention is paramount for slowing cognitive decline and reducing risk of incident dementia.

Frailty describes an age-related state of deterioration in functioning across multiple physiological systems, resulting in increased vulnerability to adverse health outcomes including falls, delirium, multimorbidity, cognitive decline, admission to hospital, and increased mortality [6–8]. It is estimated that frailty is present in millions of elderly people worldwide [9]. One of the most commonly used constructs to objectively quantify frailty is the frailty index (FI) presented by Rockwood and colleagues, a model that defines frailty as a state caused by the accumulation of health deficits during the life course [10]. Symptoms, signs, diseases, and disabilities are considered as deficits [10] and combined in FI with the ratio of deficits present to the total number of deficits considered. FI allows frailty to be regarded as gradable instead of arbitrary present or absent. Individuals with a higher FI score indicate a higher degree of frailty. Therefore, the accumulation of deficits FI model is reversible and sensitive to modification [11], as reflected by its continuous feature.

Numerous studies have been conducted to investigate the relationship between frailty and dementia in recent years [12, 13]. Data from the UK Biobank incorporated in 196,123 participants indicated that high frailty had an increased dementia risk compared with those with low frailty [HR (95%Cl) = 3.68 (3.11, 4.35)] [14]. Moreover, a longitudinal study based on the Uniform Data Set showed that among MCI patients, higher degree of frailty was associated with a higher risk of developing dementia [15]. A noteworthy feature of frailty is modifiability, which means that an individual can shift between degrees of frailty [16]. Thus, frailty is potentially reversible and provides a probable target for dementia prevention. Similarly, modifiable lifestyle factors including alcohol drinking, smoking and physical inactive have been reported to be risk factors for adverse brain outcomes [17–19] and thus are also potential targets for preventing or slowing the progression of cognitive decline [20–22]. A study based on 550 subjects enrolled in the Whitehall II cohort found that any level of alcohol consumption was associated with adverse brain outcomes including cognitive decline [17]. Moreover, a study conducted in 1,697 German elder adults reported that history of lifetime smoking was associated with increased risk of cognitive decline [18]. Among former smokers, a longer time since smoking abstinence was associated with better cognitive function with reference to current smokers [18], indicating smoking cessation to be a potential prevention for cognitive impairment. Abundant studies have also proposed the protective role of physical activity on cognitive function [23, 24]. However, none of the studies has examined the potential interactive and combined effects of frailty with lifestyle factors on cognitive function.

In this current study, we included 3,279 individuals within Dongfeng-Tongji (DFTJ) cohort, to investigate the association of frailty with cognitive function by using a 35-point cumulative deficit FI. We further tested the effect modifications of lifestyle factors on the above associations.

## Methods

### Study Populations

This study was carried out based on the Dongfeng-Tongji cohort, whose details have been previously described [25]. Briefly, the DFTJ cohort was established in 2008–2010, embedded within retirees from Dongfeng Motor Corporation (DMC) in Shiyan, China. Participants were followed up every five years through face-to-face interview. In the second follow-up visit, we recruited 3291 retired workers from two large plants of DMC to collect their cognitive function by using the mini-mental state examination (MMSE) test. In the present study, we excluded participants who were diagnosed with dementia but had normal MMSE score (*n* = 12). The left 3,279 participants were finally included in the following analysis (Fig. S1). The Ethics Committee of Tongji Medical College, Huazhong University of Science and Technology approved this study and written informed consents were provided by all participants.

### Assessment of frailty

Frailty status of the study participants was assessed based on a 35-item FI (Fig. 2), which was constructed following the standard procedure presented by Samuel D Searle and colleagues [26]. FI were calculated by using health variables obtained through questionnaires and physical examination in the second follow-up of 2018. Health variables were included for the construction of FI if they met the following criteria: age-related, not becoming universal at middle age and multidimensional in health status [26]. After screening self-reported or measured data, health variables including co-morbidity, medication usage, self-reported health problems and life quality, decline in physical performances (as measured by the activities of daily living scale), and depressive symptoms (as measured by the geriatric depression scale) were selected for construction of FI. The complete list of health variables and their coding are available in Table S1. Based on the FI distribution in this study and the literature reports [27, 28], a cut-off point of 0.15, the 75^th^ percentile of FI, was used to categorize the participants into two groups: robust (FI < 0.15) and frail (FI ≥ 0.15).

### Assessment of cognitive function

In this study, we used a Chinese version of the Mini-mental State Examination (MMSE) to evaluate the cognitive function of study participants through face-to-face questionnaire survey. MMSE is a 30-point test included tests of presentation, registration, attention and calculation, recall, language, and visual-spatial skills. One point is added for each correctly answered question. Lower scores are more indicative of impaired cognitive function. The cut-off point for the determination of cognitive impairment was 20 for individuals with 6 or less years of education, and 24 for individuals with 7 or more years of education, according to a previous study based on Chinese population [29].

### Assessment of covariates

Information on covariates including demographic characteristics (age, gender, education level, marital status) and lifestyle factors (smoking and drinking status, physical activity, etc.) were collected through face-to-face questionnaire survey. Body mass index (BMI) was generated by dividing weight in kilograms by the square of the height in meters. Education level was classified as primary school or below, junior or high school, college or above. Marital status was dichotomized into single (single, divorced, or widowed) and married. Participants who had smoked > 1 cigarettes per day for at least 1 year were defined as current smokers; those who had ever smoked and had quitted smoking ≥ 6 months were defined as former smokers; otherwise, they were defined as never smokers [30]. Individuals who had drunk alcohol > 1 time per week for at least 1 year were defined as current alcohol drinkers; those who had ever drunk and had quit drinking ≥ 6 months were defined as former alcohol drinkers; otherwise, they were defined as never alcohol drinkers [31]. Because of the low proportions of former smokers (8.9%) and former drinkers (4.7%) in the study participants, we categorized current and former smokers into ever smokers, and combined former and current drinkers into ever drinkers. Regular physical exercise was assessed on the basis of exercise duration and frequency of various exercise types. Those who exercised for at least 20 min/day over the last 6 months were considered as regular physical exercisers; if not, they were considered as non-regular physical exercisers [32].

### Statistical analysis

Covariates and individual health variables identified for inclusion in FI had less than 5% missing. Missing data in covariates and health variables were filled with median and mode by simple imputation for continuous and categorical variables, respectively. Descriptive characteristics for study participants were presented as mean (± SD) for continuous variables or percentage (%) for categorical variables. Student’s *t*-test and Chi-square test were used to test the differences of general characteristics between subjects with normal cognitive function and cognitive impairment. Multivariate generalized linear regression model was performed to estimate the association of frailty status and FI with MMSE score by calculating regression coefficient (β) and 95% confidence interval (CI). The multivariate logistic regression model was used to estimate the associations of frailty status and FI with cognitive impairment by calculate odds ratio (OR) and 95% CI. We multiplied FI by 10 (range 1–10) for easier interpretation and β and OR would represent the proportion of change for per 0.1 increase in FI score. Both models were adjusted for age (≤ 60, 61–70, 71–80, > 80) and gender in Model 1, and further adjusted for marital status, education levels, smoking status, drinking status and regular physical exercise in Model 2. The potential confounders were selected based on the literature reports [33, 34]. The restricted cubic splines (RCS) were also used to evaluate the linearity of the association between FI and cognitive function. Furthermore, we conducted stratified analysis by age (≤ 70 or > 70), gender, smoking status (never or ever), drinking status (never or ever) and regular physical exercise (yes or no) in both multivariate linear and logistic regression models. The effect modifications of smoking status, drinking status and regular physical exercise on the association of frailty status with MMSE score and cognitive impairment were tested by including a multiplicative interaction term [frailty status (robust /frail) × lifestyle factors (categorical)] into the regression models. We also evaluated the combined effects of frailty status with lifestyle factors (smoking status, drinking status and regular physical exercise) on MMSE score and cognitive impairment by using multivariate linear and logistic regression models, respectively.

A two-sided *P* < 0.05 was considered statistically significant. All statistical analyses were performed using SPSS (version 24) and R software (version 4.1.2).

## Results

### Demographic characteristics of study participants

**Fig. 1:**
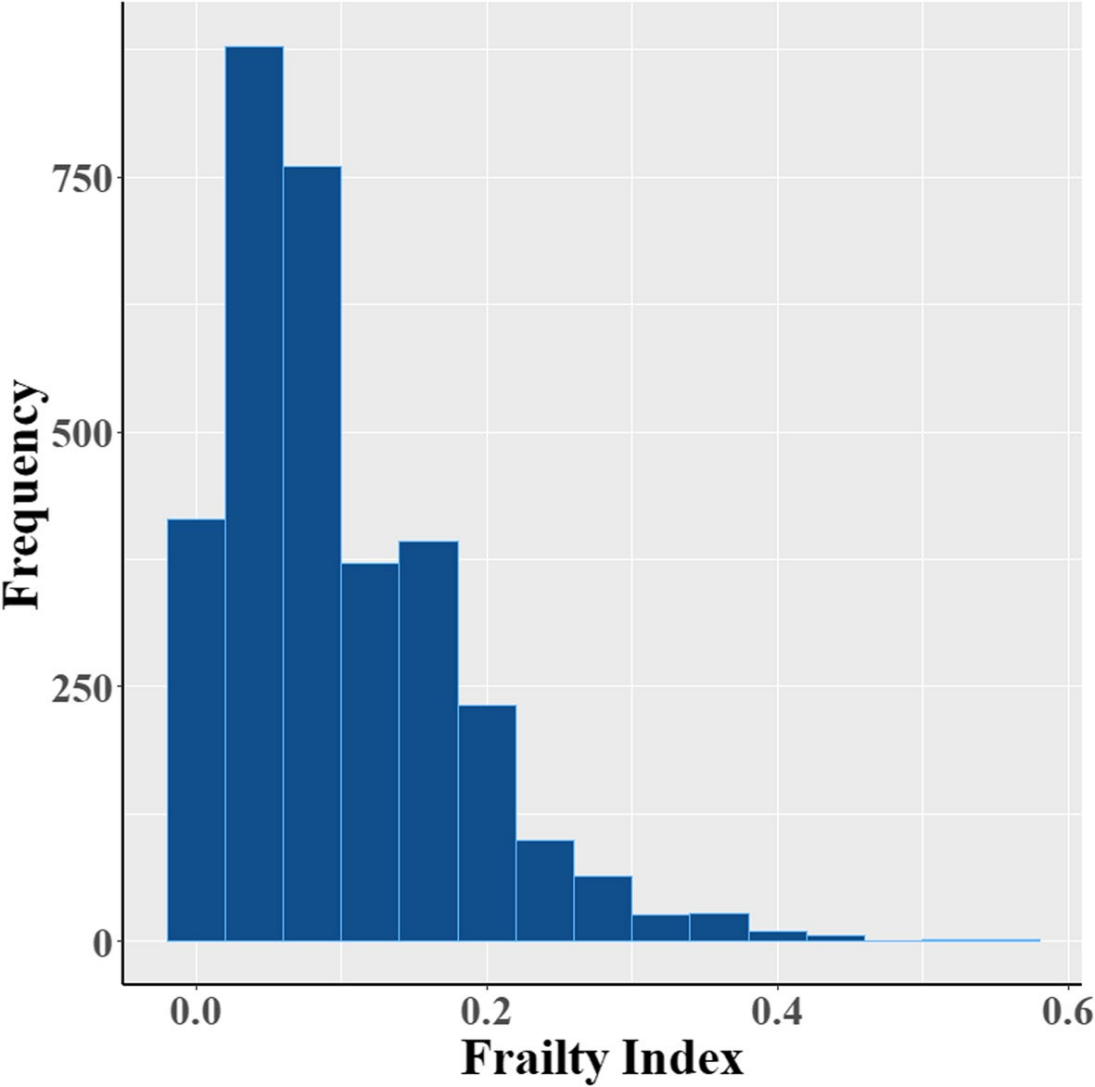
The distribution of frailty index among the study participants

The study participants had a mean [SD] age of 69.6 [8.7] years-old and a mean [SD] BMI of 23.92 [3.15]. There were 58.1% females (*n* = 1904), 78.5% never smokers, 75.9% never alcohol drinkers, and 91.2% regular physical exercisers. Consistent with the previous study [26], FI of the study subjects presented a gamma distribution with a maximum score of 0.543 (Fig. 1), which makes it possible to be compared across studies. In total, the study subjects had a mean (± SD) FI of 0.098 (± 0.078), and a mean (± SD) MMSE score of 25.77 (± 3.59). 699 (21.3%) participants met the criteria for frailty and 398 (12%) participants had cognitive impairment. Table 1 shows the distribution of descriptive characteristics for the study participants stratified by cognitive function. Compared to those with normal cognitive function, participants with cognitive impairment were more likely to be less educated, physically inactive, had higher FI and percentages of frailty (all *P* < 0.05, Table 1).

**Table 1:** Distribution of general characteristics for study participants (*n* = 3279)

Variables	Total (*n* = 3279)	Normal (*n* = 2881)	Cognitive impairment (*n* = 398)	*P*
Age, mean ± SD (years)	69.6 ± 8.7	69.4 ± 8.7	71.4 ± 9.1	< 0.001
Age, n (%)				< 0.001
≤ 60	599 (18.3)	539 (18.7)	60 (15.1)	
61–70	973 (29.7)	868 (30.1)	105 (26.4)	
71–80	1410 (43.0)	1237 (42.9)	173 (43.5)	
> 80	297 (9.1)	237 (8.2)	60 (15.1)	
Gender, n (%)				0.837
Males	1375 (41.9)	1210 (42.0)	165 (41.5)	
Females	1904 (58.1)	1671 (58.0)	233 (58.5)	
Education, n (%)				0.003
Primary school or below	1088 (33.2)	934 (32.4)	154 (38.7)	
Junior/High school	2035 (62.1)	1799 (62.4)	236 (59.3)	
College or above	156 (4.8)	148 (5.1)	8 (2.0)	
Marriage, n (%)				0.113
Single	482 (14.7)	413 (14.3)	69 (17.3)	
Married	2797 (85.3)	2468 (85.7)	329 (82.7)	
BMI, mean ± SD (kg/m^2^)	23.92 ± 3.15	23.89 ± 3.15	24.10 ± 3.18	0.235
Smoking, n (%)				0.855
Never	2575 (78.5)	2262 (78.5)	313 (78.6)	
Former	293 (8.9)	260 (9.0)	33 (8.3)	
Current	411 (12.5)	359 (12.5)	52 (13.1)	
Drinking, n (%)				0.236
Never	2489 (75.9)	2199 (76.3)	209 (72.9)	
Former	155 (4.7)	131 (4.5)	24 (6.0)	
Current	635 (19.4)	551 (19.1)	84 (21.1)	
Regular physical exercise, n (%)				0.001
No	289 (8.8)	237 (8.2)	52 (13.1)	
Yes	2990 (91.2)	2644 (91.8)	346 (86.9)	
Frailty index (FI), mean ± SD	0.098 ± 0.078	0.097 ± 0.076	0.110 ± 0.084	0.001
Frailty status, n (%)				0.008
Robust (FI < 0.15)	2580 (78.7)	2287 (79.4)	293 (73.6)	
Frail (FI ≥ 0.15)	699 (21.3)	594 (20.6)	105 (26.4)	
MMSE score	25.77 ± 3.59	26.67 ± 2.44	19.22 ± 3.76	< 0.001

*Abbreviations: BMI* Body mass index, *MMSE* Mini-mental state examination

Continuous variables are shown as mean ± standard deviation (SD)

^a^*P* values were calculated by using Student’s *t* test for continuous variables and Chi-square test for categorical variables

### Associations of frailty and cognitive function

**Fig. 2:**
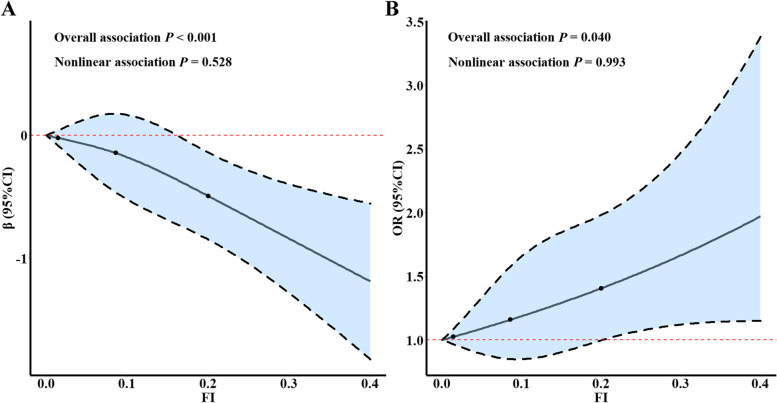
The association between FI and cognitive function based on restricted cubic spline function. **A** MMSE score. **B** Cognitive impairment. Abbreviations: FI, frailty index; MMSE, mini-mental state examination. Note: The spline plots were drawn by using 3 knots (10^th^, 50^th^, 90^th^ percentiles). Models were adjusted for age, gender, marriage, education level, smoking status, drinking status and regular physical exercise

As shown in Table 2, compared with the robust group, frail participants had a lower MMSE score [β (95%CI) = -0.56 (-0.86, -0.27)] and a higher odds of cognitive impairment [OR (95%CI) = 1.30 (1.02, 1.66)] (Model 1). Such associations slightly attenuated after further adjustment for marital status, education levels, smoking status, drinking status and regular physical exercise [β (95%CI) = -0.49 (-0.76, -0.21) for MMSE score and OR (95%CI) = 1.29 (1.00, 1.65) for cognitive impairment] (Model 2). With per 0.1 increase in FI, there was 0.36 decrease in MMSE score (95%CI: -0.51, -0.20) and 1.20-fold increase in the odds of cognitive impairment (95%CI: 1.05, 1.36) (Model 1). After further adjustment for marital status, education levels, smoking status, drinking status and regular exercise, for each 0.1 increase in FI, there was a 0.28 decrease in MMSE score (95%CI: -0.43, -0.13) and a 1.19-fold odds of cognitive impairment (95%CI: 1.04, 1.35) (Model 2) (Table 2). We further tested the dose–response relationship shapes between FI and cognitive function with the RCS models. Spline regression analysis showed significant linear associations of FI with both MMSE score and cognitive impairment (both *P* for overall association < 0.05, and *P* for nonlinear association > 0.05) (Fig. 2A and B).

**Table 2:** Associations of frailty with cognitive function among the study participants

Frailty	Events/n	MMSE score		Cognitive impairment	
		**β (95% CI)**	***P***	**OR (95% CI)**	***P***
***Model 1***					
Frailty status ^a^					
Robust	293/2580	reference		reference	
Frail	105/669	-0.56 (-0.86, -0.27)	< 0.001	1.30 (1.02, 1.66)	0.034
FI ^b^	398/3279	-0.36 (-0.51, -0.20)	< 0.001	1.20 (1.05, 1.36)	0.007
***Model 2***					
Frailty status ^a^					
Robust	293/2580	reference		reference	
Frail	105/669	-0.49 (-0.76, -0.21)	0.001	1.29 (1.00, 1.65)	0.045
FI ^b^	398/3279	-0.28 (-0.43, -0.13)	< 0.001	1.19 (1.04, 1.35)	0.011

*Abbreviations: FI* Frailty index, *MMSE* Mini-mental state examination

Model 1: adjusted for age (≤ 60, 61–70, 71–80, > 80), gender; Model 2: further adjusted for marriage status, education level, smoking status, alcohol drinking status, and regular physical exercise

^a^Robust: frailty index < 0.15; Frail: frailty index ≥ 0.15; ^b^Per 0.1 increased in frailty index

### Stratified analysis of frailty status and cognitive function

**Fig. 3:**
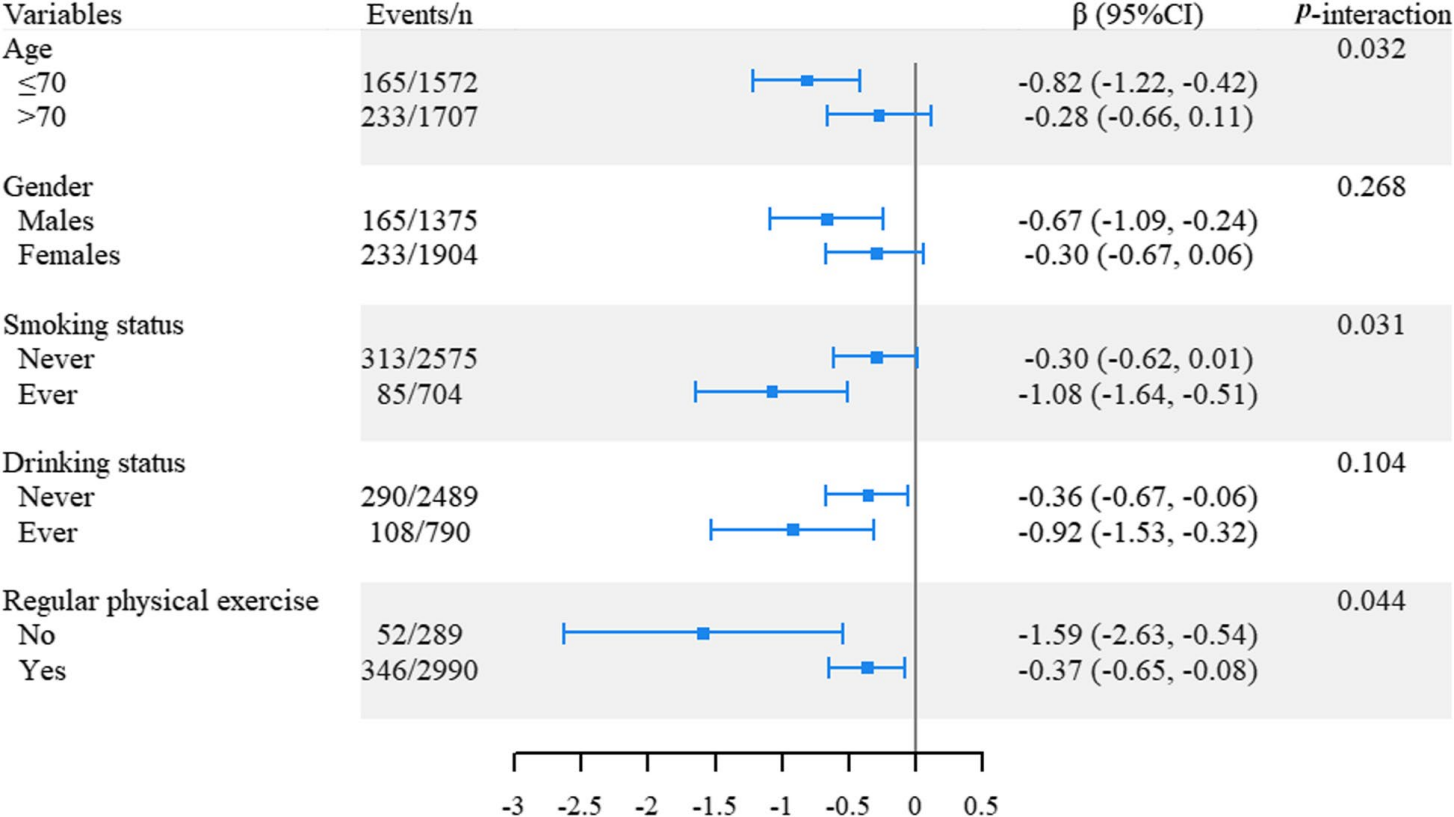
Stratified analysis for the association of frailty status with MMSE score. Abbreviations: MMSE, mini-mental state examination. Note: Models were adjusted for age, gender, marriage, education level, smoking status, drinking status, and regular physical exercise

In the stratified analysis for the association between frailty status and MMSE score, the association remained significant in each subgroup by age, gender, smoking status, drinking status and regular physical exercise. Moreover, we observed the significant effect modifications of age, smoking and physical activity on the above association (*P* for interaction = 0.032, 0.031 and 0.044, respectively). Specifically, the effect of frailty status on lower MMSE score was obviously shown among individuals aged ≤ 70 [β (95%CI) = -0.82 (-1.22, -0.42), *P* < 0.001], ever smokers [β (95%CI) = -1.08 (-1.64, -0.51), *P* < 0.001] and physical inactive individuals [β (95%CI) = -1.59 (-2.63, -0.54), *P* = 0.003], but was significantly attenuated among individuals aged > 70 [β (95%CI) = -0.28 (-0.66, 0.11), *P* = 0.161], never smokers [β (95%CI) = -0.30 (-0.62, 0.01), *P* = 0.059] and physical active subjects [β (95%CI) = -0.37 (-0.65, -0.08), *P* = 0.011]. However, we did not find the significant effect modifications of gender, and alcohol drinking status on the above association (all *P* for interaction > 0.05) (Fig. 3).

As for cognitive impairment, we did not find significant interactions of age, gender, physical exercise, smoking and drinking status with frailty. However, the effect of frailty status was predominately shown for subjects who were aged ≤ 70 [OR (95%CI) = 1.59 (1.07, 2.37), *P* = 0.023], males [OR (95%Cl) = 1.51 (1.04, 2.18), *P* = 0.030], ever smokers [OR (95%Cl) = 1.93 (1.17, 3.18), *P* = 0.010], ever alcohol drinkers [OR (95%Cl) = 1.63 (1.01, 2.61), *P* = 0.044] and physical inactive individuals [OR (95%Cl) = 2.45 (1.21, 4.94), *P* = 0.013] (Fig. S2).

### Combined effects of lifestyles with frailty on cognitive function

**Fig. 4:**
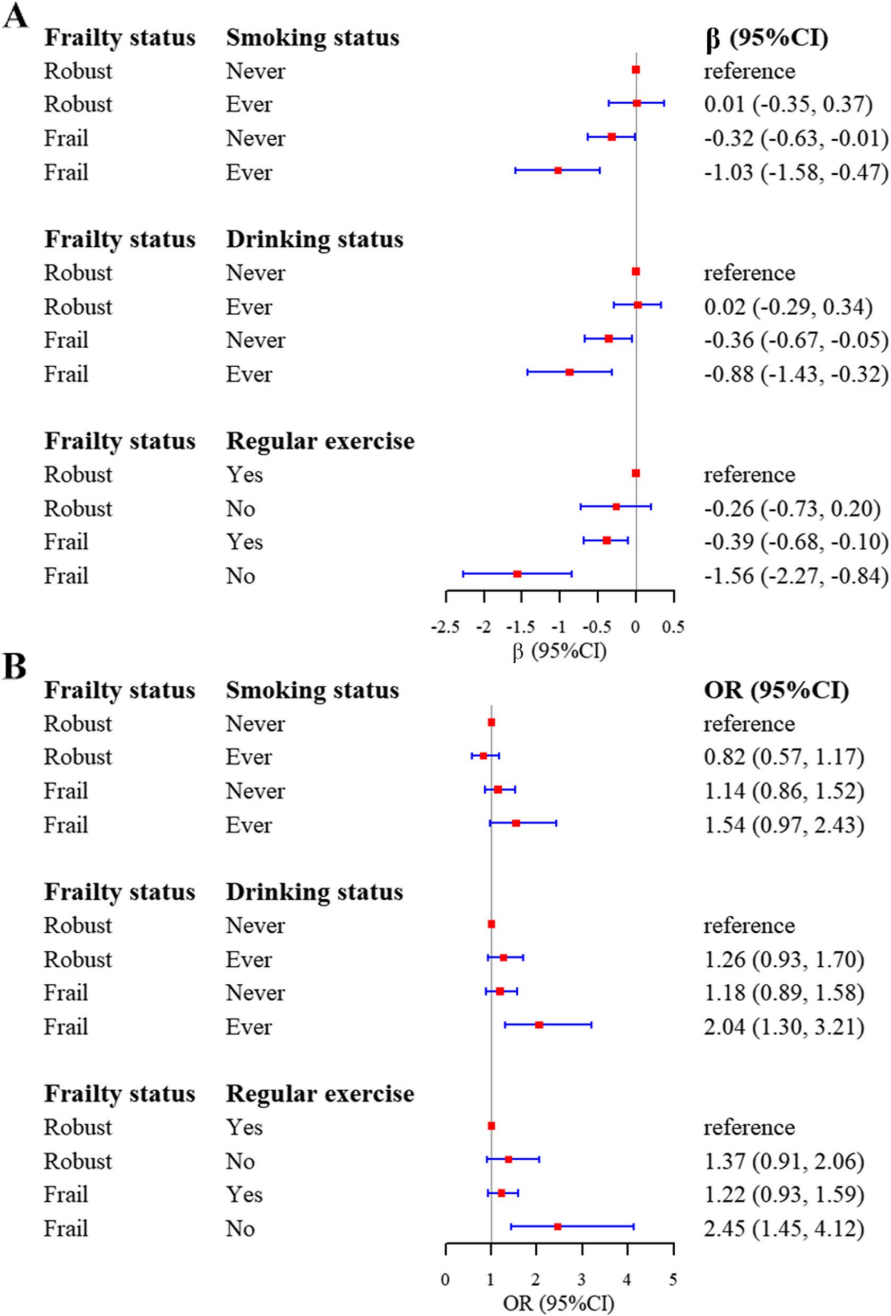
Combined effects of frailty status with lifestyles on cognitive function. **A** MMSE score (**B**) Cognitive impairment. Abbreviations: OR, odds ratio; MMSE, mini-mental state examination. Note: Models were adjusted for age, gender, marriage, education level, smoking status, drinking status, and regular physical exercise

We illustrated the combined effects of frailty status with lifestyle factors including dichotomous smoking status, drinking status and regular physical exercise. It is shown that, compared to never smokers with a robust status, frail individuals and ever smokers were more likely to have a lower MMSE score [β (95%Cl) = -1.03 (-1.58, -0.47), *P* < 0.001] and a higher odds of cognitive impairment [OR (95%Cl) = 1.54 (0.97, 2.43), *P* = 0.065]. Compared to never alcohol drinkers with a robust status, those within the frail and ever drinkers group had a lower MMSE score [β (95%Cl) = -0.88 (-1.43, -0.32), *P* = 0.002] and a higher odds of cognitive impairment [OR (95%Cl) = 2.04 (1.30, 3.21), *P* = 0.002]. Frail and physical inactive individuals had a lower MMSE score [β (95%Cl) = -1.56 (-2.27, -0.84), *P* < 0.001] and a higher odds of cognitive impairment [OR (95%Cl) = 2.45 (1.45, 4.12), *P* = 0.001] compared with the robust and physical active group (Fig. 4A and B).

## Discussion

In this cross-sectional study, we found that frailty, as measured by a 35-item frailty index, was significantly associated with decreased MMSE score and increase odds of cognitive impairment. Furthermore, the associations between frailty status with MMSE score were stronger among individuals aged ≤ 70, ever smokers and physical inactive individuals. Significant effect modifications were observed for age, smoking and regular physical exercise on the above association. More importantly, our findings extended the previous literatures by describing the combined effects of frailty, smoking, alcohol drinking and physical inactive on cognitive impairment.

The present findings about the association between frailty and cognitive function were consistent with previous cross-sectional and longitudinal evidence where frailty was estimated based on either continuous FI or categorised frailty status. A cross-sectional study based on 3,497 older Japanese adults found significant relationships between frailty and MCI [OR (95%CI) = 2.0 (1.5, 2.5)] [35]. Furthermore, a prospective study embedded within 7,439 U.S. community-dwelling older adults showed that frail individuals had lower level (-0.03SD/year, 95%CI: -0.04, -0.01) and steeper decline (-0.01SD/year, 95%CI: -0.012, -0.005) in global cognitive function than the robust [8]. In addition, a longitudinal study incorporated 14,490 participants aged 50 years and older from the National Alzheimer’s Coordinating Centre found that per 0.1 increment in FI was associated with elevated risk of developing MCI [HR (95%CI) = 1.66 (1.55, 1.78)] [36]. In this study, we incorporated both frailty measures: FI and dichotomised frailty status, which allows our results to be compared across a wider range of related studies. Additionally, while FI measures the degree of frailty in each individual and quantitatively summarize vulnerability, the analysis based on categorical frailty status could contribute to the identification of high-risk population at cognitive impairment. Our findings provide comprehensive evidence for the relationship of frailty and cognition among Chinese populations by using a multidimensional accumulation of deficits index. Further longitudinal studies are needed to elucidate the result.

We observed that the associations of frailty status with a lower MMSE score and higher odds of cognitive impairment were more evident among physical inactive individuals, but were significantly attenuated among subjects with regular physical exercise. Together with the effect modification of physical activity on the association between frailty status and MMSE score, our findings suggest that regular physical exercise may attenuate the frailty induced cognitive impairment. The result is consistent with previous clinical trials in elderly people with frailty syndrome [37, 38]. A randomized clinical trial embedded within Spanish community-dwelling geriatrics found that a supervised-facility multicomponent exercise program could reverse frailty and improve cognitive functionality. A 9% increase in the MMSE score was observed in the intervention group, whereas the control group saw a maintained or even slightly lost in global cognitive function (*P* = 0.025) [37]. In a recent systematic review and meta-analysis of 10 randomized trials, Rossi and colleagues evaluated the effects of physical exercise on the cognition of community-based senior adults with frailty syndrome [38]. They found that the intervention group displayed a significant increase in global cognition of 2.26 points, as measured by MMSE, compared to the control groups (mean difference = 2.26; 95% CI, 0.42 – 4.09; *P* = 0.02) [38]. Despite some studies showed that physical exercise only improved domain-specific cognitive performance instead of global cognitive function [39], the present study is generally consistent with the previous studies in this field. Several potential biological explanations for the protective effect of physical exercise on cognition proposed by previous studies are the facilitation of neuroplasticity [40], maintenance of brain volume [41], reduction of chronic stress, inflammation and oxidation [42].

Similar effects were also found for smoking status. We observed that the inverse associations between frailty status and cognition were stronger in ever smokers while weaker or not significant in never smokers, and smoking could enhance the association between frailty status and MMSE score. Few evidence reported the effects of smoking in the association between frailty and cognition, however, studies have proposed that smoking was a risk factor for cognitive decline [43] as well as a predictor for developing frailty [44–46]. A study included 1,079 non-demented Chinese participants and found that cigarette smoking was associated with both poorer global cognition and tau pathologies [43]. Moreover, a systematic review based on 5 studies provided the evidence of smoking as a predictor of worsening frailty status among community-dwelling middle-aged and older people [45]. In a longitudinal study of 2,542 elderly people in England aged 65 or over, Kojima and colleagues also reported that current smokers were more likely to develop frailty compared with non-smokers [OR (95%CI) = 1.60 (1.02, 2.51)] [46]. Interventions of smoking cessation are able to effectively halt cognitive decline among elderly people, which was found by a clinical trial with a 24-month follow-up [44], is also parallel with our findings. Therefore, the current evidence suggests that there might be a potential difference in the frailty-cognition association among never and ever smokers. Further research into its difference is critical to shed light on the result. The results also addressed the importance of smoking cessation on preventing cognitive impairment especially among the frail.

We found significant combined effects of frailty status with unhealthy lifestyles including smoking, alcohol drinking, and physical inactive on cognitive impairment. Abundant evidence has shown that lifestyle factors including smoking, alcohol drinking and physical exercise were associated with cognitive function. In a systematic review and meta-analysis of 243 observational prospective studies and 153 randomized controlled trials, Yu and colleagues reported that smoking, physical exercise, and frailty were evidence-based interventions for AD prevention [47]. Although substantial heterogeneity exists in the observed associations between alcohol consumption and cognitive impairment, various studies reported adverse effects of alcohol consumption on cognitive function, giving evidence on the importance of alcohol abstention for dementia prevention [48, 49]. A longitudinal population-based cohort study among 2,416 participants reported that heavy alcohol drinking (> 1.2 oz of ethanol consumption per day) in the midlife (average age = 52 years) could increase the risk for MCI in the later life (average age = 87 years) and decrease the age at incidence [48]. A Mendelian randomization study based on publicly available datasets also found that alcohol consumption was associated with an earlier age of AD onset [49]. Moreover, lifestyle factors including smoking, increased alcohol intake and physical inactive are well-established modifiable risk factors for frailty [9], which substantially overlap with those proposed for dementia. Given the associations between lifestyle behaviours and frailty as well as the proposed frailty-cognition link, we may hypothesise that frailty not only impact cognitive function through independent pathways but also act as a mediator between lifestyle factors and cognition, which has already been examined by a previous study [14]. Unhealthy lifestyles might exert a notable proportion of their adverse effects on cognitive function through an associated increment in degree of frailty, in other words, by slowing the rate of health deficits accumulation. Our result indicated the public health implication of lifestyle interventions on cognitive protection.

Previous studies had proposed several potential underlying mechanisms for association between frailty and cognitive function but there is no concurrence yet. One of the possible intrinsic connection between frailty and increased risk of dementia may be an underlying increased risk of stroke and cerebrovascular disease [50]. Healthy lifestyles including physical exercise, smoking and drinking cessation may prevent the onset of cardiovascular events and lower the risk of cognitive impairment. Other potential mechanisms including chronic inflammation, mitochondrial dysfunction, DNA methylation, hypothalamic–pituitary–adrenal axis dysfunction, AD pathology, hormone, nutrition, and mental health [12, 13], may also underline the above associations but need further investigations.

In this study, we used a composite cumulative deficit frailty index to evaluate the degree of frailty. Compared with the phenotypic frailty definitions [51] described in previous studies, FI could depict frailty in a more multidimensional manner by including physical, psychological, and social support items [12]. There were some limitations of the present study. Firstly, there is a lack of objective physical measurements (gait speed, griping strength, etc.) and laboratory tests (routine blood examination, brain MRI, cerebrospinal fluid biomarkers, etc.) in assessment of frailty [52]. Secondly, the healthy worker effect in this study may cause underestimation in prevalence of frailty and the observed frailty-cognition association. Finally, the cross-sectional study design is insufficient to infer causality. More longitudinal studies that are based on cumulative burden index model and regularly screened for MMSE score are warranted to validate these observations.

## Conclusion

In conclusion, we found significant associations between frailty and cognitive impairment, and these effects could be attenuated by never smoking and regular physical exercise. We also pointed out the combined effects of frailty and lifestyle factors including smoking status, drinking status and regular physical exercise on cognitive impairment. Together, our findings proposed the susceptibility of the frail to cognitive impairment and the public health implication of lifestyle interventions on dementia prevention, especially among the frail.

## Supplementary Information

**Additional file 1: Figure S1.** Flow chart of the study. **Table S1.** Factors used for frailty index calculation. **Figure S2.** Stratified analysis for the association of frailty status with cognitive impairment.

## Data Availability

The datasets used and/or analysed during the current study are available from the corresponding author on reasonable request.

## Abbreviations

MCI: Mild cognitive impairment
AD: Alzheimer’s disease
DALYs: Disability adjusted life years
FI: Frailty index
DFTJ: Dongfeng-Tongji
DMC: Dongfeng Motor Corporation
MMSE: Mini-mental state examination
BMI: Body mass index
SD: Standard deviation
CI: Confidence interval
OR: Odds ratio
RCS: Restricted cubic spline
DNA: Deoxyribonucleic acid
MRI: Magnetic resonance imaging
