# Supplementary material for: Frailty and its combined effects with lifestyle factors on cognitive function: a cross-sectional study
Source: BMC Geriatr. 2023 Feb 6;23:79. doi: 10.1186/s12877-023-03761-0 (PMC9900934; doi:10.1186/s12877-023-03761-0)
Supplement: Supplementary file 1 — Additional file 1: Figure S1. Flow chart of the study. Table S1. Factors used for frailty index calculation. Figure S2. Stratified analysis for the association of frailty status with cognitive impairment. [file 12877_2023_3761_MOESM1_ESM.docx]

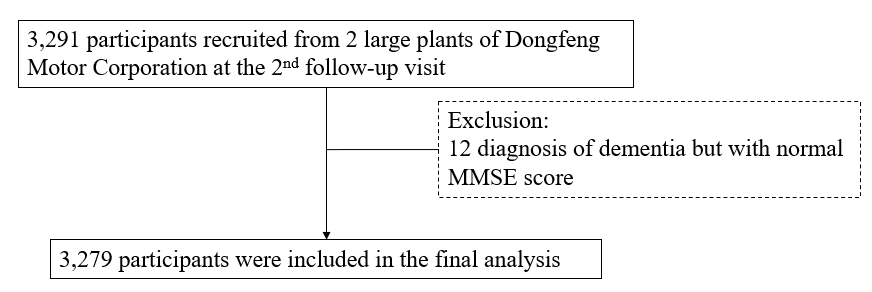


**Figure S1.** Flow chart of the study.

**Table S1**. Factors used for frailty index calculation.

| 35 variables included in the frailty index | Cut point |
| --- | --- |
| 1. Hypertension | Yes=1, No=0 |
| 1. Hyperlipidemia | Yes=1, No=0 |
| 1. Diabetes | Yes=1, No=0 |
| 1. Coronary Heart Disease | Yes=1, No=0 |
| 1. Myocardial Infarction | Yes=1, No=0 |
| 1. Stroke | Yes=1, No=0 |
| 1. Emphysema | Yes=1, No=0 |
| 1. Tumour | Yes=1, No=0 |
| 1. PD | Yes=1, No=0 |
| 1. Osteoporosis | Yes=1, No=0 |
| 1. Feel Tired | Most of time=1, Sometime=0.5, Rarely=0 |
| 1. Declined food intake during the last 3 months | Severe decrease=1, Moderate decrease=0.5, No decrease=0 |
| 1. Weight loss during the last 3 months | weight loss greater than 3kg=1, does not know=0.5, weight loss less than 3kg=0 |
| 1. BMI | ＜18.5 or ≥28=1; 24-28=0.5; 18.5-24=0 |
| 1. Has suffered psychological stress or acute disease in the past 3 months | Yes=1, No=0 |
| 1. Lives independently | Yes=0, No=1 |
| 1. Takes more than 3 prescription drugs per day | Yes=1, No=0 |
| 1. Self-rating of health in comparison of other people of the same age | not as good=1, does not know=0.5 , as good or better=0 |
| 1. Fecal incontinence | Yes=1, No=0 |
| 1. Urinary incontinence | Yes=1, No=0 |
| 1. Help grooming | Yes=1, No=0 |
| 1. Help using toilet | Yes=1, No=0 |
| 1. Help eating | Yes=1, No=0 |
| 1. Help moving | Yes=1, No=0 |
| 1. Help walking | Yes=1, No=0 |
| 1. Help dressing | Yes=1, No=0 |
| 1. Help up stairs | Yes=1, No=0 |
| 1. Help bathing | Yes=1, No=0 |
| 1. Feel life is empty | Yes=1, No=0 |
| 1. Get bored | Yes=1, No=0 |
| 1. Be in good spirits most of time | Yes=0, No=1 |
| 1. Feel happy | Yes=0, No=1 |
| 1. Feel helpless | Yes=1, No=0 |
| 1. Have more problems with memory than most | Yes=1, No=0 |
| 1. Feel hopeless | Yes=1, No=0 |

**Abbreviations:** BMI, body mass index; PD, Parkinson’s disease.


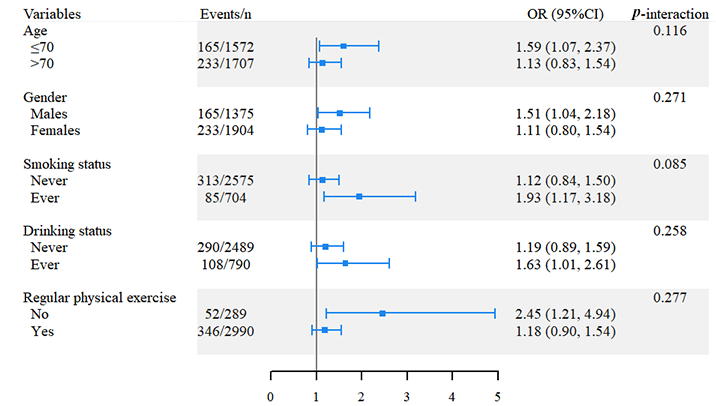


**Figure S2. Stratified analysis for the association of frailty status with cognitive impairment.**

**Note:** Models were adjusted for age (≤60, 61-70, 71-80, >80), gender, marriage, education level, smoking status, drinking status, and regular physical exercise.
